# Identification of stress from incremental dentine stable isotope trends using linear enamel hypoplasia

**DOI:** 10.1371/journal.pone.0359651

**Published:** 2026-10-09

**Authors:** Emma Smith, Angela Lamb, Linda Fibiger, Mario Šlaus, Anita Adamić Hadžić, Catriona Pickard

**Affiliations:** 1 School of History Classics and Archaeology, University of Edinburgh, Edinburgh, United Kingdom; 2 National Environmental Isotope Facility, British Geological Survey, Nottingham, United Kingdom; 3 Croatian Academy of Sciences and Arts, Zagreb, Croatia; University of Otago, NEW ZEALAND

## Abstract

High-precision sampling of dentine serial increments facilitates analysis of short-term variations in stable isotope ratios. Temporal resolution of micro-sampled increments is sufficiently high that the impact of short-term periods of physiological stress, which have well-described effects on stable isotope ratios, may be captured. However, distinguishing the effects of diet vs physiological effects on δ^13^C and δ^15^N is non-trivial. This study presents a comparative analysis of one manifestation of physiological stress, linear enamel hypoplasia (LEH), and incrementally sampled dentine stable isotope ratios. Incidence and age at formation of LEH of 32 permanent canines from two medieval Croatian populations (13^th^–16^th^ centuries CE cemeteries at Dugopolje and Udbina) were recorded. The canines were subsequently micro-sampled for δ^13^C and δ^15^N measurement. Intra-observer tests of δ^13^C and δ^15^N scatterplot interpretation were undertaken to investigate consistency in the identification of stress vs diet effects on stable isotope ratio trends. A strong relationship was found between stress-related variation in stable isotope trends and LEH. It is therefore proposed here that evaluation of teeth for LEH, and where identified, the recording of age of formation, become a standard component of incremental dentine stable isotope analysis pre-treatment protocols to improve stress identification in serially sampled dentine stable isotope ratio trends to enhance interpretations of past diet and individual isotope biographies.

## Introduction

Stable isotope analysis of serial sections of dentine collagen is a valuable tool for investigating diet in early life. This method provides information about average diet over a period of months to a few years depending on both the tooth selected and the size of increments taken. The temporal resolution of this approach is sufficiently high to allow periods of physiological stress to be discerned. However, identifying stress from stable isotope trends alone is not straightforward, and as a consequence, this can confound interpretation of diet and the construction of life histories [1].

Here, we present the results of an investigation into the issues associated with identifying physiological stress in serial incremental isotope data, and also offer suggestions for better understanding this complex and uncertain phenomenon to improve the accuracy of interpretation. Further, we consider the intersection of stress, and breastfeeding and weaning, on stable isotope profiles during infancy. This is evaluated through comparison of the carbon (δ^13^C) and nitrogen (δ^15^N) stable isotope ratios of incremental sections of dentine and the formation ages of linear enamel hypoplasias (LEH), a sign of physiological stress, in canine teeth.

### Context and background

Stable isotope analysis has long been used to elucidate past dietary behaviours [e.g., 2–5]. Stable isotope ratios of collagen, extracted from archaeological bone or tooth dentine, are measured by mass spectrometry. Consumer collagen stable isotope ratios reflect those of their dietary constituents. Interpretations of diet must account for the predictable fractionation of the stable isotope ratios of food sources by the consumer, i.e., there is an increase in stable isotope ratio values between food and consumer, which is often referred to as the diet-consumer offset [6].

Studies of diets in past populations have typically analysed collagen carbon (δ^13^C, ^13^C/^12^C) and nitrogen (δ^15^N, ^15^N/^14^N) stable isotopes ratios [5,7,8]. δ^13^C is a proxy for plant type consumed, i.e., C_3_ vs C_4_ plants [2]. These plants have different photosynthetic pathways, and as a consequence, C_4_ plants (e.g., maize, millet, and sugarcane) typically have higher δ^13^C values than C_3_ plants (e.g., wheat, barley, and rye) [9]. While marine plants overwhelmingly utilize the C_3_ photosynthetic pathway, the carbon sources of oceanic plants are isotopically distinct from those of terrestrial C_3_ plants resulting in marine C_3_ plants having higher δ^13^C than terrestrial specimens [10,11]. Nitrogen stable isotope ratios may distinguish between the contribution of these two food webs to diet. δ^15^N is a proxy for dietary protein sources, discriminating between plant-based diets and those that include animal protein, and terrestrial from marine foods [3]. δ^15^N values are typically higher in marine foods than terrestrial resources due to the more complex food web of the former (i.e., more trophic levels with fractionation of ^15^N at each step) [9,12].

Early studies of palaeodiet relied on visual parsing or linear mixing modelling to reconstruct diet from stable isotope ratios [e.g., 7, 8, 13, 14]. The working theory underpinning these models was generally one of direct routing of protein from diet to collagen (i.e., only dietary protein atoms contributed to bone or dentinal collagen). While the principles of stable isotope analysis are largely unchanged since its initial inception, technical and theoretical advances have been made. For example, better understanding of human diet-consumer fractionation effects suggested that the offset was higher for nitrogen, at c. + 6‰ [15], than the previously suggested c. + 3 to +5‰ [e.g., 16]. Furthermore, advances in Bayesian analysis and the wide availability of modelling software allows for multi-macronutrient routing to be taken into account, so that the contribution of carbon atoms from the protein, carbohydrate and lipid components of diet to collagen synthesis is reflected in dietary models [6,17]. Improvements in instrumentation permit measurement of very small collagen masses (e.g., 0.2–0.3 mg) – allowing for the analysis of minute serial sections of dentine, providing the pathway for high resolution analysis of individual diets [18–20].

As there is no turnover of primary dentine and it forms in a predictable, largely steady and unidirectional manner, it provides a permanent record of isotopic signatures incorporated during tooth development [18,21]. Dentine stable isotope values can be used to evaluate diet in early life, potentially elucidating the weaning process [e.g., 18, 22–24]. Due to the high resolution of incremental data, both long- and short-term changes can be observed. Dentine development, which is under strong genetic control is more resistant than bone to exogenous and environmental effects that can slow or delay tissue synthesis [25,26]. This resilience allows for periods of stress, which may not be evident in bone, to be recorded in dentine. Physiological stress that results in nutritional deficiency leads to the breakdown of body tissues for reuse in new tissue synthesis [27]. Due to the nature of dentine secretion, stress-related signals may be isotopically visible, which, while valuable for further assessing individual life histories, can complicate dietary investigation. Stress-related changes to stable isotope values are usually identified by increased δ^15^N values, the result of further fractionation during tissue recycling [28,29]. King *et al.* [1] proposed that a quantitative threshold can be used to identify stress during weaning, and suggested that a change in δ^15^N of greater than two standard deviations (2σ) above the maternal mean could be applied to distinguish stress from dietary change during infancy. This assumes dietary consistency before and after episodes of stress; however, stress may be coincident with changes to diet, e.g., during weaning (see Fig 1), which may obscure the expected changes. Decreased δ^13^C may also indicate stress; carbon atoms may be recycled from body fat stores, which have lower δ^13^C values than proteinaceous tissues [27,28]. The influence of stress on collagen δ^13^C values, however, is less consistent than on δ^15^N. Clinical studies have shown no consistent pattern in δ^13^C response to stress. Mekota *et al.* [28] and Neuberger *et al.* [27] found that δ^13^C values decreased with anorexia-induced stress, while Canterbury *et al.* [30] found no change or increased δ^13^C in post-operative bariatric patients. This is likely because while nitrogen is sourced only from dietary protein or recycled proteinaceous tissues, collagen carbon may be derived from multiple sources – dietary proteins, carbohydrates, and lipids, as well as recycled tissues – the complex interplay of which, arguably, accounts for variation in observed δ^13^C changes in response to stress [e.g., 27, 30]. This implies that stable isotope trends alone should not be the *sole* method on which stress identification hinges.

**Fig 1:**
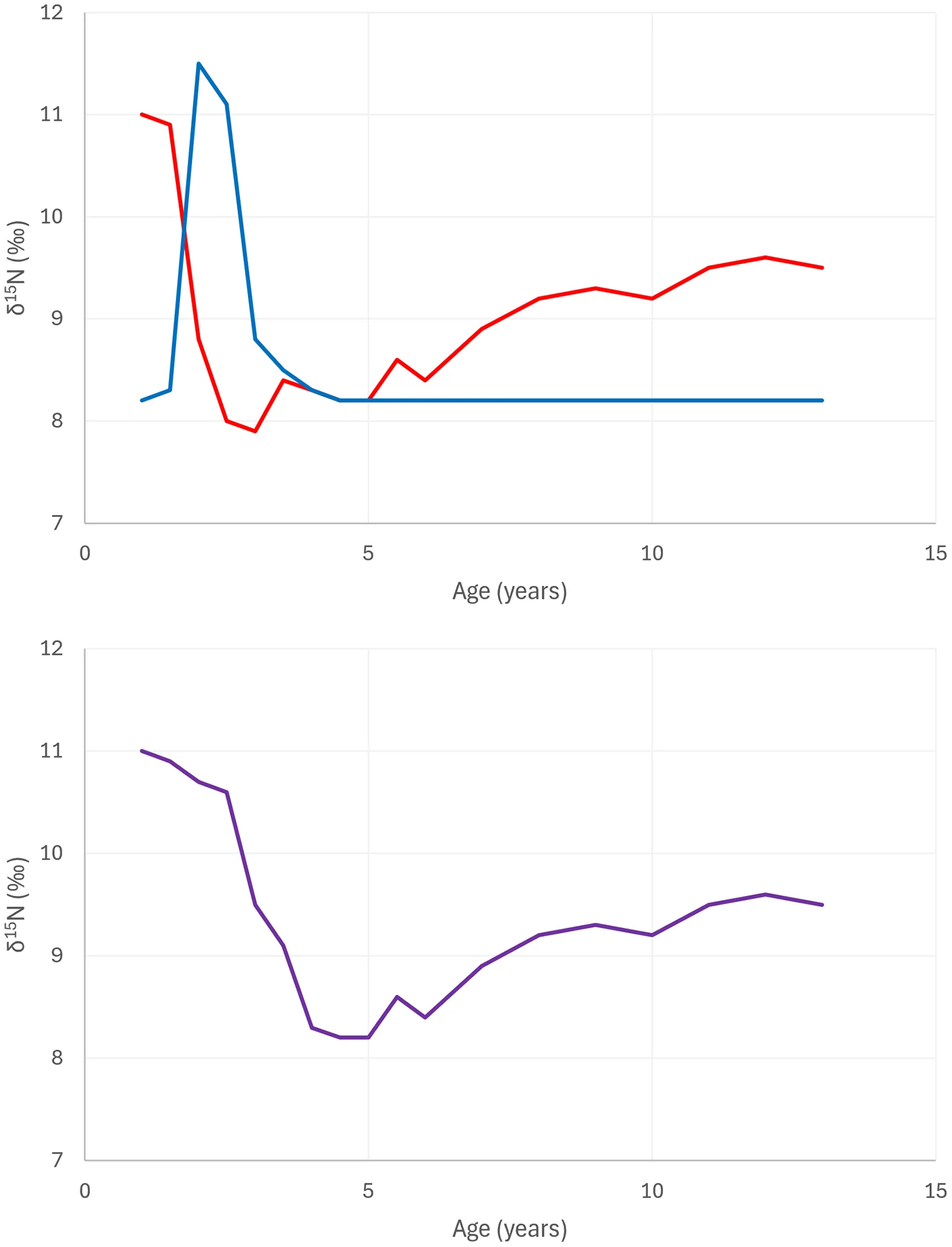
Example data. The red line in the top image shows a weaning curve isolated from stress, while the blue line shows a stress peak in isolation. The bottom image shows these in (approximate) combination, an example of how co-occurring diet and stress signals may produce an attenuated signature. This would possibly impact interpretations (e.g., weaning age). NOTE: this is for illustrative purposes only; after, as an example, Fuller et al.[21], Nitsch et al. [22], and Beaumont and Montgomery [28] but no specific stable isotope measurements were used from these sources.

Diet and stress are often interlinked. During dietary transitions such as weaning, the opposing effects of diet and stress may produce attenuated stable isotope trends (Fig 1). Reduction in breastfeeding during weaning decreases δ^15^N values; however, weaning is a stressful period, which may drive δ^15^N up [27,31]. The interaction of these effects could potentially result in a ‘flat’ or ‘drawn out’ trend, the effects of stress potentially keeping δ^15^N values elevated for a period after the weaning has been completed. Taken at face value such a trend could be interpreted as ‘late’ weaning, somewhere between 4 and 5 years old, however, without the presence of stress an age of c. 2.5 years may be given for end of weaning (Fig 1). Consequently, while stress may not always be apparent in isotope values, its impacts on stable isotope trends can influence interpretations of diet, weaning, and other physiological changes.

### Linear enamel hypoplasia as an indicator of childhood stress

LEH is a deficiency in enamel thickness, macroscopically visible as a band (but also seen as pitting) around the tooth (Fig 2) [32–34]. This is caused by a disturbance to amelogenesis during the matrix phase, when the enamel framework is laid down prior to full mineralisation [21,35]. LEH is commonly considered to be an indicator of nutritional stress but can result from tooth infection or trauma [32]. The cause can be distinguished by considering the formation times and the spread of LEH throughout the dental arcade. In metabolic aetiologies, LEH should be evident on multiple teeth and have overlapping formation times. Anterior teeth (incisors and canines) have a greater affinity for the development of LEH than posterior teeth (particularly the molars), which is likely related to differing enamel morphology and development [32].

**Fig 2:**
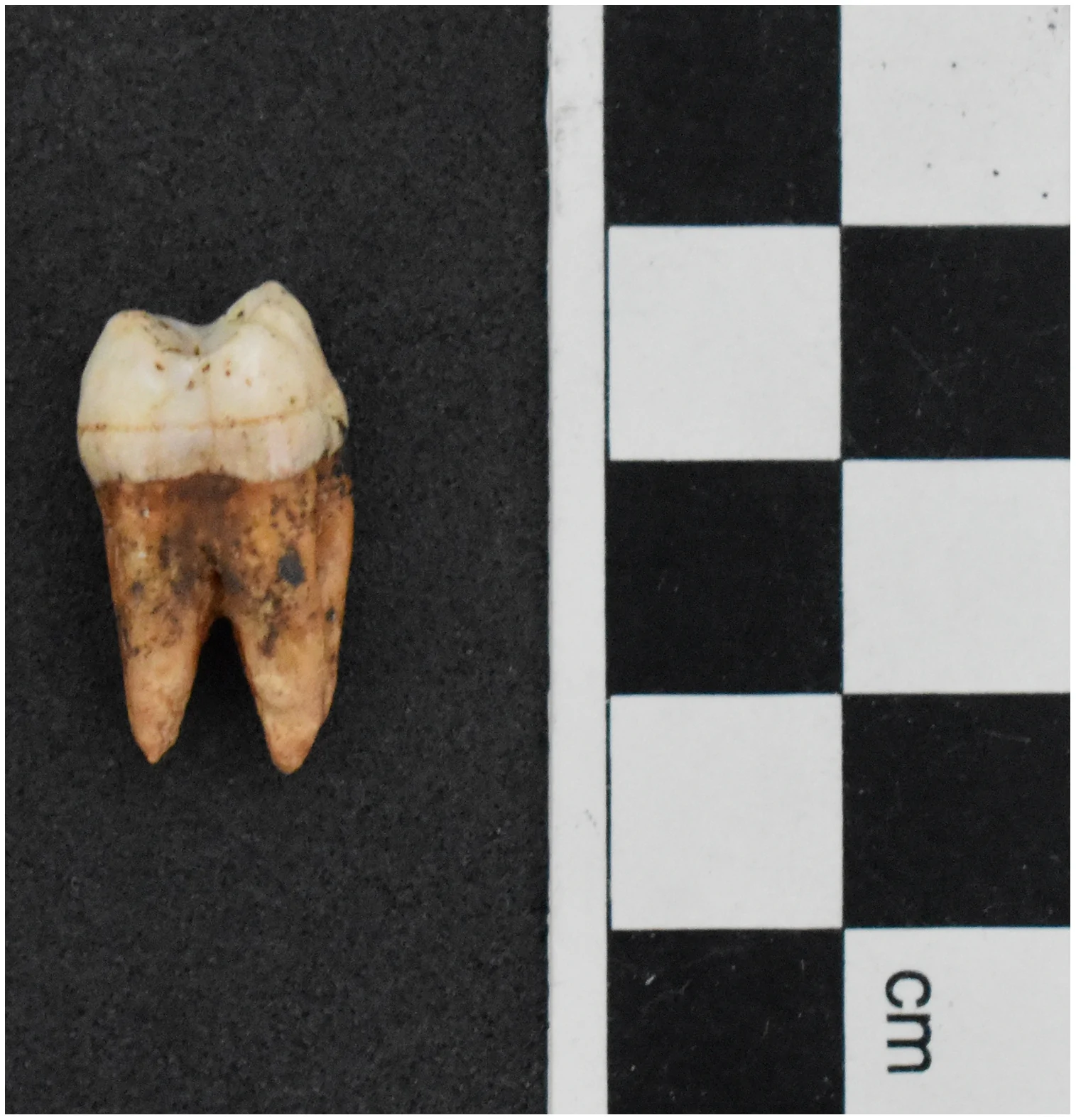
Molar with band of LEH. Photograph taken by author.

Like dentine, the matrix phase of enamel development is continuous and relatively uniform, which permits formation ages to be assigned to identified bands of LEH [36–38]. The ability to age both LEH and dentine increments allows direct comparison of the physical manifestation of physiological stress with stable isotope trends. While there is variation (genetically and environmentally caused) in enamel and dentine development and it cannot be said with complete certainty they are uniformly co-forming, the resolution at which LEH formation age is calculated, and the size of the incremental samples, would most likely obscure any differences [18,36,38,39]. While it is difficult to be precise about the potential delay between the stress and formation of LEH, ameloblasts are sensitive to physiological-based disruption and several studies have found associations between the formation of LEH and known stress periods (such as weaning) so it is likely coeval, or closely coeval [35].

A time lag between the experience of stress and its manifestation in dentine collagen isotope values has been postulated. Experimental studies of mammal dentine appear to support this assertion – abrupt change in diet is associated with a gradual shift in dentine collagen stable isotope ratios, the result of gradual turnover of the metabolic pool of nutrients [40]. However, Balasse *et al*. [40] noted that the equilibration period of dentinal collagen isotope ratios may actually be rapid, and that the observed gradual change may be an artefact of sampling procedure. Horizontal increments or punches do not capture individual dentine deposition layers [18], which are curved (see Fig 1, Curtis *et al*. [41]). Incremental dentine stable isotope ratios, therefore, reflect a ‘weighted’ time average of diet and other physiological influences.

### Isotopic stress and LEH

Smith [42] compared the relationship between serial incremental dentine isotope trends and LEH in a small number of individuals from the Medieval–Early Modern population from Whitefriars Cemetery, Perth, Scotland. One individual (Sk524), with four LEH formation periods (and overlapping formation times of multiple LEHs recorded across the whole dental arcade), exhibited increased δ^15^N at three out of four bands (Fig 3). In comparison, another individual (Sk471) from Whitefriars with no visible LEH exhibited no change in δ^15^N (Fig 3). Analysis of individuals from medieval St. Mary Spital Cemetery, London, found likely stress-related increases in incremental dentine δ^15^N in association with LEH, and in some instances with inverse co-variation of δ^13^C (see SRP98–30920 and SRP98–20682, figs 5 and 6 in Walter *et al.,* [43]). However, in two 4^th^ to 7^th^ century CE individuals from Transylvania, Romania, (see ArchM69 and ArchM73 in fig 5, Crowder *et al.* [44]) no isotopic signs of stress were observed in association with LEH. In each of the studies discussed above, the sample sizes were very small. The aim of this study, therefore, is to expand the sample size to allow for statistical exploration and to potentially validate observations of association between stress-related changes to stable isotope trends and LEH.

**Fig 3:**
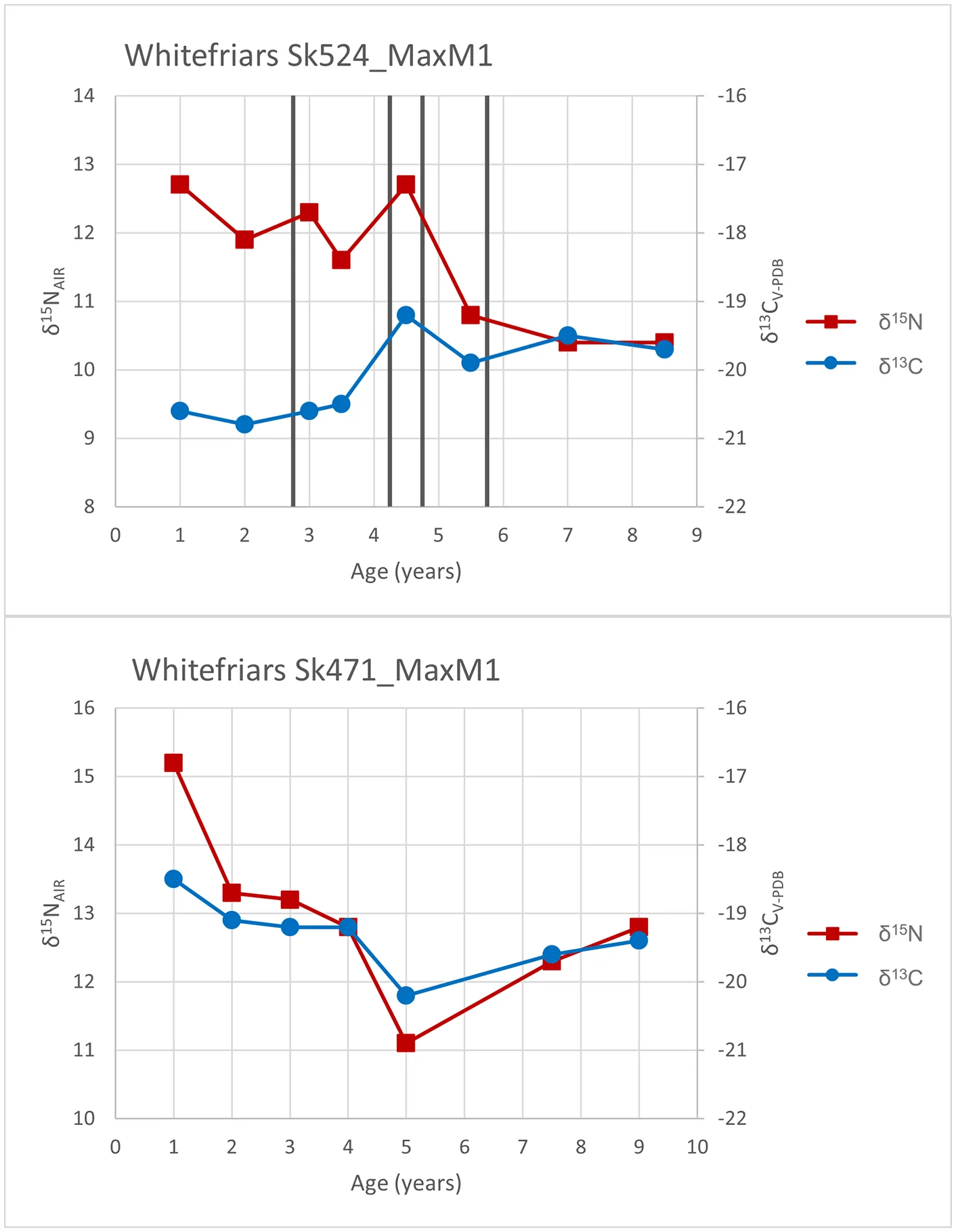
δ^13^C and δ^15^N data from two juvenile individuals from the medieval/early-modern Whitefriars populations, Perth, Scotland. LEH was identified on Sk524 (shown as black vertical bars) while no LEH was recorded on Sk471.

Garland *et al.* [45] compared the formation ages of Wilson bands with incremental dentine stable isotope values in early colonial period (CE 1605–1680) individuals from St. Catherine’s Island, USA. Wilson Bands, a microdefect in enamel with similar aetiology to LEH, are observable in histological sections [32,45]. While enamel defects were found to be more frequent during periods identified as weaning there were no obvious signs of physiological stress from the isotope data alone. Wilson Bands may suggest a less severe stress than the presence of LEH, and therefore, might be expected to have little impact on stable isotope trends.

### Archaeological background

Two contemporaneous but socially distinct populations from 13^th^ to 16^th^ century CE Croatia were used in this study. These collections are curated by the Croatian Academy of Sciences and Arts in Zagreb. Diet and age at weaning is described briefly here to contextualize the identification of physiological stress.

Dugopolje-Vučipolje-Crkvine (DVC), the cemetery site of a rural agricultural population, used between the 13^th^ to 16^th^ centuries CE, is located in the Adriatic Hinterland of Croatia (Fig 4) [46–48]. Diet, modelled from stable isotope ratios, was relatively homogenous. The population subsisted largely on C_3_ resources with greater reliance on plant-based than terrestrial animal foods, and with a minor but important contribution from C_4_ resources. The plant crops likely included cereals such as wheat, rye, and millet, as well as fruit [46,49,50]. The incremental dentine isotopic data suggests that the proportion of C_3_ vs C_4_ food sources in diet varied throughout childhood – it is not known if this variability continued into adulthood due to the time-averaged nature of bone collagen isotope values. There is no isotopic evidence from either bone or dentine collagen for marine or freshwater fish in diet. Weaning was likely completed between 2.5 and 3.5 years.

**Fig 4:**
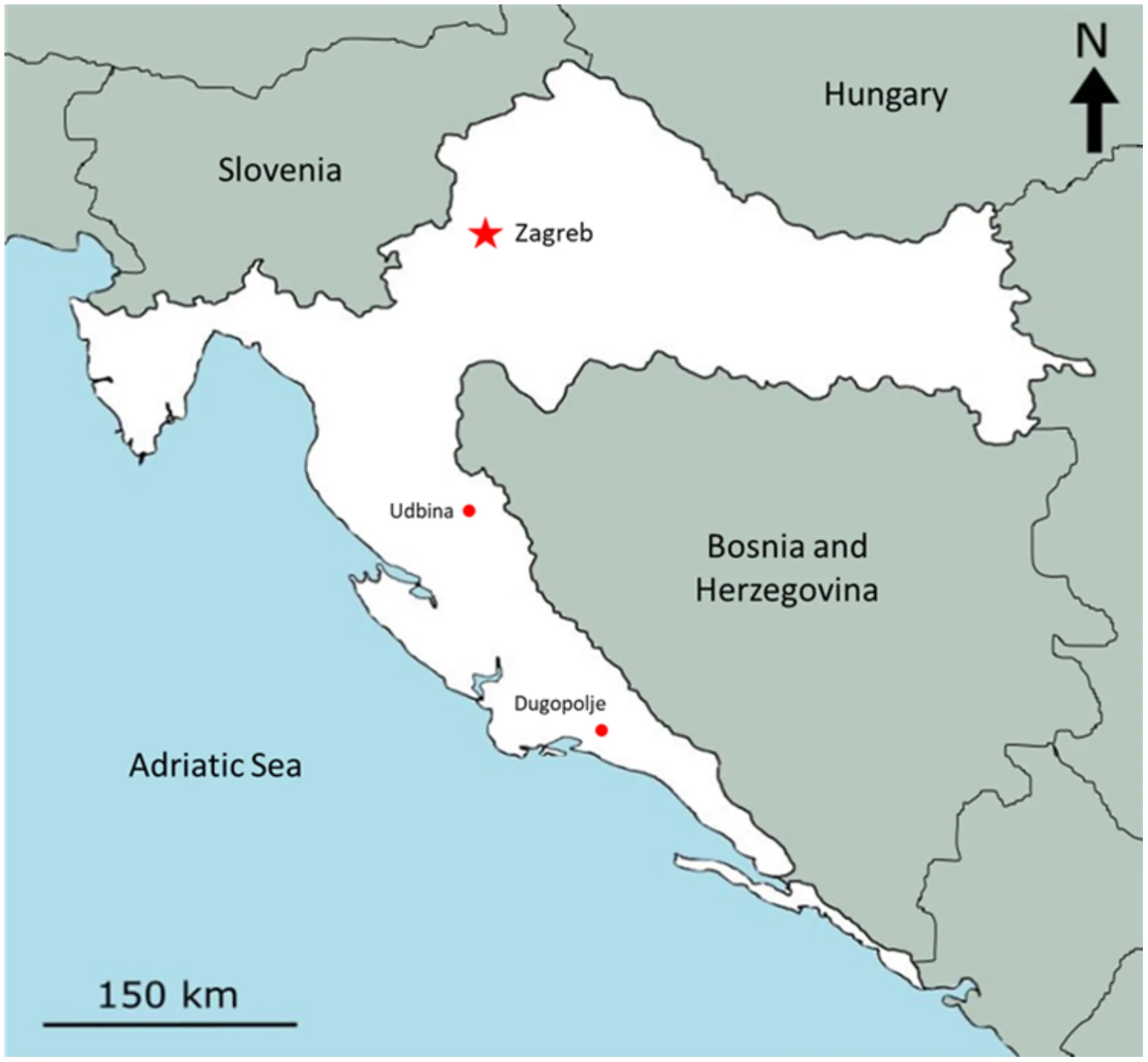
Map of Croatia showing locations of Udbina and Dugopolje.

Udbina Sveti Jakov (USJ), also situated in the Adriatic Hinterland, lies 150 km to the northwest of Dugopolje. The USJ cemetery population is likely of higher status than the individuals from DVC, based on the quality of grave goods, which included coins and jewellery, and also the socio-economic importance of the site – Sveti Jakov Cathedral was the seat of the Bishop of Krbava prior to 1460 CE [51]. Many Croatian nobles were known to have lost their lives at the nearby Battle of Krbava Field in CE 1493 and at least some of the dead are thought to have been buried at USJ [48,51–54]. Diet at USJ was predominantly C_3_-based, however, δ^15^N values were consistently higher than those from DVC, suggesting higher proportions of animal products in diet. C_4_ food source contributions to adult diet at USJ was variable and may relate to sugarcane consumption or perhaps higher consumption of products from animals foddered on C_4_ resources. While variation in C_3_ vs C_4_ contributions to diet are also evident at USJ during childhood this is less pronounced than at DVC. Fish was a small component of some diets. The estimated weaning ages from incremental dentine stable isotope data of seven individuals from USJ suggested completion between 2.0 to 4.0 years.

## Materials and methods

Teeth from thirty-two individuals from Dugopolje (n = 15) and Udbina (n = 17) were analysed. Individuals were selected based on the presence of at least two non-adjacent, relatively unworn anterior teeth including at least one canine. The majority of individuals selected had visible LEH (n = 30), with additional individuals (n = 2) selected to act as ‘non-LEH’ controls. Ethical approval for this work was granted by the University of Edinburgh’s School of History, Classics and Archaeology Ethics Committee. All necessary permits were obtained for the described study, which complied with all relevant regulations.

### Recording of linear enamel hypoplasia

For the purpose of this research, the field method (i.e., macroscopic rather than microscopic) of LEH identification was conducted for three reasons:

1. Presence and Severity of Stress: microscopically visible LEH (e.g., Wilson Bands) are unlikely to have resulted from stress severe enough to have a notable impact on stable isotope ratios [32,55].
2. Age Estimation Precision: aging LEH formation to within a few days by counting perikymata is an unnecessary level of precision where incremental dentine sections represent months to years [e.g., 56].
3. Applicability of Method: it is intended that the method be widely applicable, with no specialised microscopy equipment required [e.g., 35].

LEH measurement was carried out using sliding callipers under a bright light source. Crown height was measured from the centre of the labial side cemento-enamel junction to the apex of the crown; then, from this same point the distance to each band of observed LEH was measured (see Goodman and Song [35] for protocol). These measurements were carried out by one of the authors (ES) and repeated four weeks later to assess intra-observer error. Measurements were also carried out on a subset of the sampled individuals (see S1 Table) by an independent ‘external’ observer as both an additional intra-observer test (with measurements again carried out at four-week intervals), and to assess inter-observer error. The external observer holds an MSc in Human Osteoarchaeology and has c. 1 year of experience recording skeletal pathology but is not experienced in the measurement of LEH age-at-formation. Intra- and inter-observer analyses are an important part of osteological analysis [e.g., 57–59] – and particularly important in the investigation of LEH due to the potential for both variation in the identification of the LEH bands and in the measurement of the CEJ-LEH distance for formation age. The LEH formation ages assigned by each observer were compared statistically using a Wilcoxon signed-rank test for both the intra- and inter-observer tests.

Formation age of the LEH bands was assigned using The London Atlas to create a modified deciles approach [38], selected here over the more commonly used deciles approach [36], to ensure a robust comparison between LEH and incremental dentine isotopic trends. The age range of the crown (4.9 and 4.6 years for the maxillary and mandibular canines, respectively) was divided by 10 to find the age range of each of the deciles; this was then compared to the measured bands of LEH to identify was decile each belonged to thus find the estimated formation age ranges.

### Collagen extraction and incremental sampling of dentine

For incremental sampling and stable isotope measurement, canines (maxillary or mandibular) were selected from each individual both due to their propensity for the development of LEH and the long developmental period (c. 0.6 to 14.5 years for the maxillary canine, and 0.9 to 13.0 years for the mandibular canine). The dentine growth layers of canines have a greater inclination than molars [60], this may blur the temporal resolution but using Czermak *et al.*’s [61] biopsy punch method should help mitigate the potential resolution issues. Dentine increments were also aged using the London Atlas [38], following Czermak *et al.*’s [61] suggestion to use each anatomic region (e.g., crown and root) to help account for variation in the growth rates.

Teeth were set in Herculite 2 (5:2 ratio of Herculite:water) with the labial surface of the tooth facing upward to hold the teeth in place while cutting. Herculite was used (as opposed to another medium such as resin) as it could be removed by submerging in water. Once set, a 2–3 mm longitudinal section of the tooth was cut using an Isomet Precision Saw. Sections were then demineralised in 0.5 N HCl. Once demineralised the tooth sections were sampled from crown to root tip using a 1 mm biopsy pen following Czermak *et al.* [61] with each biopsy punch sample put into a pre-labelled vial. It was noted which samples belonged to the crown and which to the root. The incremental punches were then freeze dried. Some increments were combined with adjacent ones to ensure suitable collagen weights, providing a total of 499 increments of dentine collagen for stable isotope measurement. Stable isotope measurements of the dentine increments were carried out at the NERC National Environmental Isotopes Facility at the British Geological, using a Thermo Finnigan EA IsoLink coupled to a Delta V Plus isotope ratio mass spectrometer via a Conflo IV interface (further details are supplied in the S1 File).

### Identification and Statistical Analysis of Stress

As this research investigates the relationship between LEH and stable isotope ratios, we focus here on the dentine stable isotope values of the crown portion of the canine (representing c. 0.6/0.9 to 5.5 years of age). Isotope trends were initially assessed for conventional signs of stress, i.e., increase in δ^15^N accompanied by decrease in δ^13^C [27,29].

Following identification of possible stress-related changes to stable isotope values, the correlation between these isotopic changes and LEH was analysed statistically, using chi-squared tests with post-hoc Cramér's V for effect size. Using these tests, the data was evaluated in two ways:

1. **Individual Stress-LEH Correlation Assessment:** A comparison of dentine isotope trends that were identified as either ‘with’ or ‘without’ evidence for stress versus presence or absence of LEH. The individuals were categorised as with stress if this was suggested by changes in δ^15^N and δ^13^C at any point across the whole scatterplot. This comparison was not dependant on age assignment or coeval presence of LEH.
2. **Division Stress-LEH Correlation Assessment:** A more direct comparison of periods of isotopically identified stress with the presence/absence of LEH. This was achieved by dividing the incremental dentine scatterplots of the crown region of each tooth into three sections and marking each section for the presence/absence of identifiable isotopic stress and comparing this to the presence/absence of LEH across the three sections (Fig 5). This was carried out in two ways:
  - Conventional: where standard markers for stress identification from stable isotopes (i.e., increased δ^15^N) were used but δ^13^C was not evaluated as it has been shown to be variable in clinical trials and the study populations have variable C_4_ food source inclusion in diet making δ^13^C stress-related changes unreliable without context.
  - Context Integrated: this is a much more subjective analysis where the observer’s knowledge of diet and dietary variation within the populations (i.e., contextual information) was integrated into the identification of stress-related changes to stable isotope trends, i.e., not just using ‘conventional’ changes to identify possible stress but using dietary knowledge of the populations to help identify areas where stress may be present but not immediately obvious.

**Fig 5:**
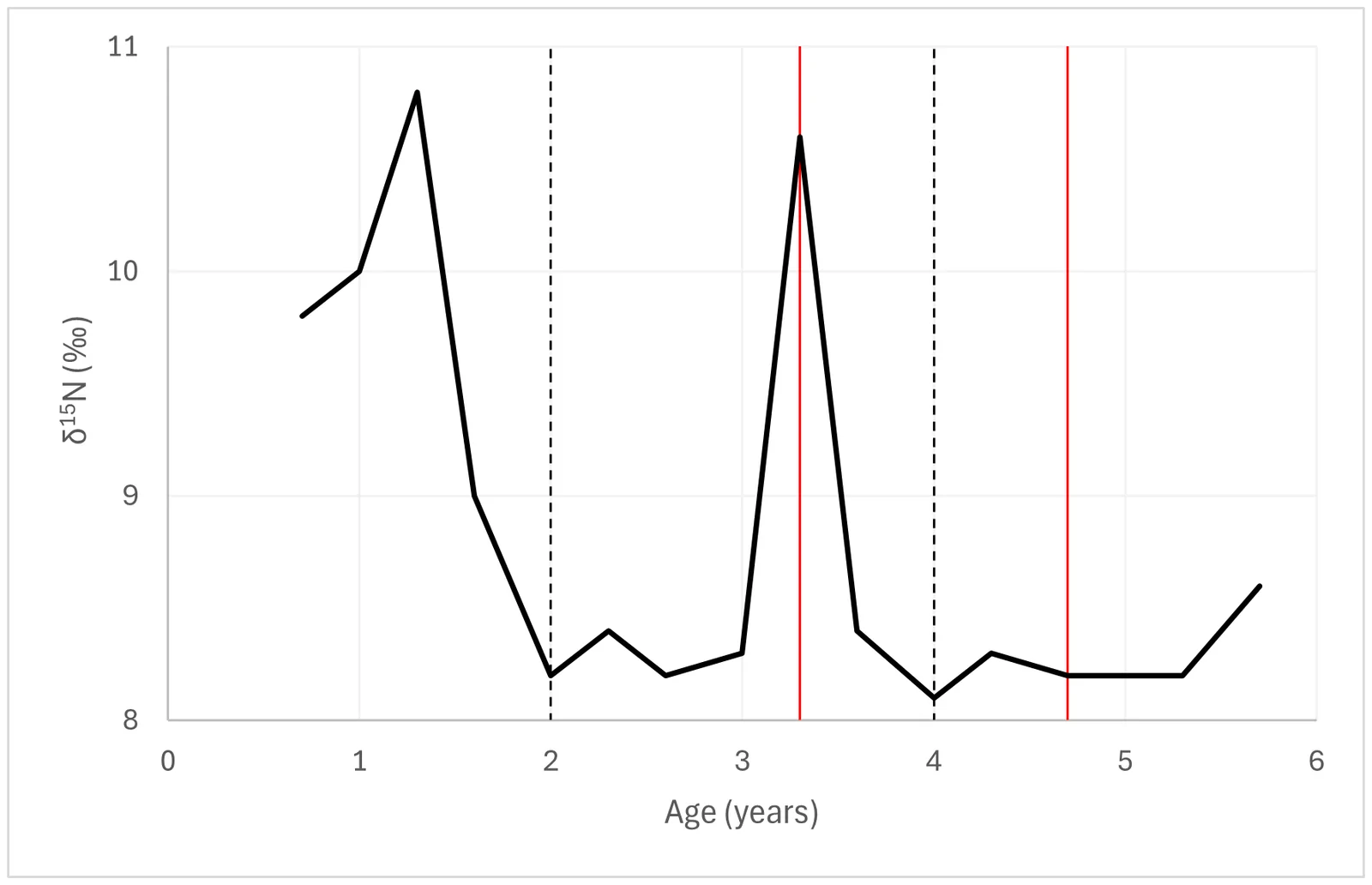
Example data with vertical dashed lines showing the divisions of the crown into three sections. The red lines show position of LEH bands. For the division stress-LEH assessment, section 1 would be marked as having isotopic stress but no LEH; section 2 as isotopic stress with LEH; and section 3 as no isotopic stress but with LEH. NOTE: this is for illustrative purposes only.

Bands of LEH that were in the same division or could not be easily distinguished as separate events were grouped as one for this comparison.

The instances where there was an association between observer-identified stress in a stable isotope scatterplot and presence of LEH was evaluated using the data from the **Division Stress-LEH Correlation Assignment**. Two evaluations were made:

1. Instances where areas of the scatterplots were identified by the observer as showing stress-related changes and were subsequently found to be associated with LEH (termed here **Stress-LEH Association**).
  $$\begin{array}{c} \frac{Identified\;Isotopic\;Stress\;Associated\;with\;Bands\;of\;LEH}{All\;Identified\;Isotopic\;Stress} \end{array}$$
2. Instances where the age at which bands (or groupings of bands) of LEH occurred were found to be areas on the stable isotope scatterplots identified by the observer as showing stress-related changes (termed here **LEH-Stress Association**).
  $$\begin{array}{c} \frac{Identified\;Isotopic\;Stress\;Associated\;with\;Bands\;of\;LEH}{Bands\;of\;LEH} \end{array}$$

Instances are reported as percentages.

## Results

### Observer identified periods of stress in stable isotope data

Areas of the scatterplots identified by the observer as exhibiting stress-related changes often showed increases in δ^15^N values (e.g., DVC132 and USJ199, Figs 6a and 6b). While changes in δ^13^C were also identified as potentially stress-related by the observer, the availability of C_4_ foods at DVC and USJ and the variable reliance on these resources in childhood makes the use of δ^13^C values for identifying stress unreliable in this context. Other trends that the observer identified as stress-related included ‘shallow’ weaning curves (e.g., DVC55A and USJ10A, Figs 6c and 6d), large and isolated dips in δ^13^C value (particularly notable at USJ, e.g., USJ38 and USJ15, Figs 6e and 6f), and ‘interruptions’ to a weaning curve (e.g., DVC17 and USJ170, Figs 6g and 6h).

**Fig 6:**
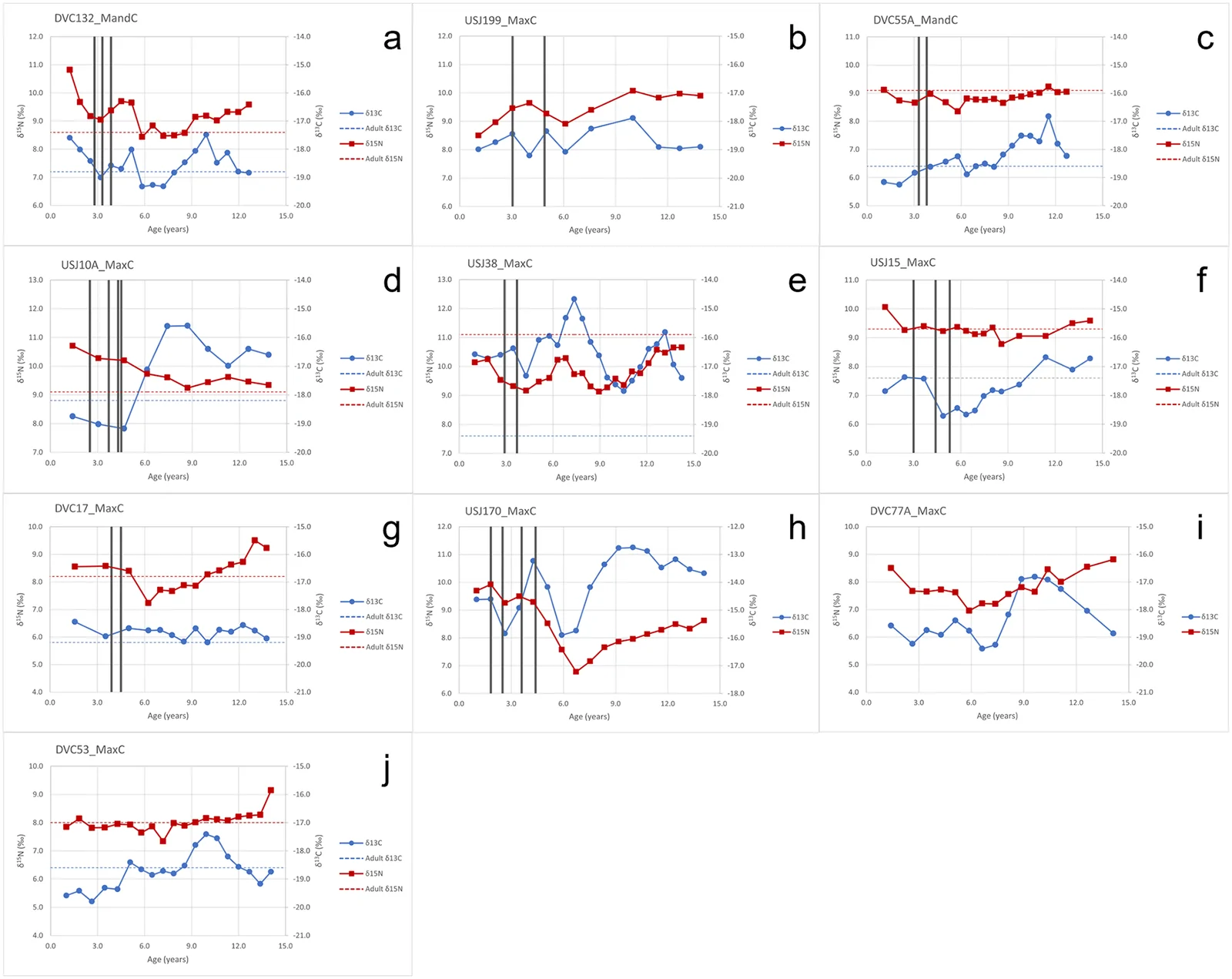
Incremental dentine stable isotope data overlaid with LEH formation times. Scatterplots of δ^13^C (blue circles) and δ^15^N (red squares) incremental data with bands of LEH in black. Where available adult rib stable isotope data is shown in dotted lines. Figs 6i and 6j show ‘control’ individuals (DVC77A and DVC53) who had no identified LEH.

Two individuals showed no signs of LEH, and no stress related changes were identified. DVC77A (Fig 6i) shows a decline in δ^15^N, likely associated with weaning, and notably has a similar δ^15^N profile to the Whitefriars individual without LEH (Sk471, Fig 2). The stable isotope ratio trends of DVC53 (Fig 6j) do not exhibit any clear indication of weaning, presenting with a flat δ^15^N profile, growth (decreasing δ^15^N) may also further obscure early weaning, i.e., prior to the period visible in the canines, by pulling the already declining δ^15^N down further giving the flat profile. The remaining graphs are presented in the S1 Fig.

### Statistical analysis of observer identified stress and LEH

**Individual Stress-LEH Correlation Assessment:** There was a total of 32 individuals with incremental stable isotope ratio data; 30 of these individuals had LEH, and of these individuals, stress was identified in 24 of them from the stable isotope ratio trends (Table 1). A strong relationship between the presence of stress-related changes to stable isotope trends and LEH is evident, but the higher p-value suggests the sample size may be too small to detect whether the relationship is meaningful (effect size = 0.68, p-value = 0.05897): the small number of counts (n = 32) make the estimates poor and thus the results are not reliable. Further, the small number of controls (n = 2) may impact the outcome of this test.

**Table 1:** Associations of LEH and stress-related changes to stable isotope trends of incremental dentine data at an individual level (Individual Stress-LEH Correlation Assessment).

		Stable Isotope Stress Identified	
		Y	N
**LEH Present**	Y	24	6
	N	0	2

**Division Stress-LEH Correlation Assessment:** To address whether there is a correlation between LEH and stress-related changes to stable isotope ratio trends more directly, the crown areas of each tooth were divided into three approximately equal sections (this varied slightly as it was guided by LEH spacing and the temporal resolution of the increments), giving 96 regions across the 32 individuals (Fig 4). Each of these divisions were marked for the presence of LEH and the presence of stress-related changes in stable isotope trends. This was first done (Test 1) using conventional identification methods, i.e., increased/increasing δ^15^N (Table 2). The relationship between LEH and stress-related changes to stable isotope trends was found to be strong (effect size (Cramér’s V) = 0.5012, p-value = 3.101e-06). A subsequent test (Test 2a) was carried out in which the observer integrated dietary knowledge of the populations into identifications of stress; this test was repeated (Test 2b) to assess intra-observer variation. The results of Test 2a (Table 3) demonstrated a strong, meaningful relationship between LEH and stable isotope trends (effect size = 0.6308, p-value = 2.489e-09). A similar distribution was found in the repeat test, Test 2b (Table 4), and the results were also found to have a strong relationship (effect size = 0.6715, p-value = 1.901e-10). The division-based stress assessments can be seen in S2 Table.

**Table 2:** Test 1: Conventional Identification -– Associations of LEH and stress-related changes to stable isotope trends of incremental dentine data based on divisions to the profiles of the crown of the incremental data (Division Conventional Stress-LEH Correlation Assessment).

		Stable Isotope Stress Identified	
		Y	N
**LEH Present**	Y	20	26
	N	1	49

**Table 3:** Test 2a: Integrated Context Identifications -– Associations of LEH and stress-related changes to stable isotope trends of incremental dentine data based on divisions to the profiles of the crown of the incremental data (Division Integrated Context Stress-LEH Correlation Assessment).

		Stable Isotope Stress Identified	
		Y	N
**LEH Present**	Y	29	17
	N	2	48

**Table 4:** Test 2b: Integrated Context Identifications -– Associations of LEH and stress-related changes to stable isotope trends of incremental dentine data based on divisions to the profiles of the crown of the incremental data (Division Integrated Context Stress-LEH Correlation Assessment).

		Stable Isotope Stress Identified	
		Y	N
**LEH Present**	Y	33	12
	N	4	47

In the division-based test, Test 2a (see Table 3), 31 areas of stress were identified from stable isotope trends by visual parsing. Of these, 29 instances were associated with LEH (**Stress-LEH Association** – 94%). In the repeated test, Test 2b (see Table 4), 37 areas of isotopic stress were identified and 33 of these were associated with LEH (89%).

Forty-five of the divisions contained LEH (as identified by ES). In Test 2a, 29 instances where LEH co-occurred with isotopic stress were identified by the observer (**LEH-Stress Association** – 64%). In Test 2b, 33 instances where LEH were associated with isotopic stress were identified (**LEH-Stress Association** – 73%).

### Collagen quality

Of 499 increments of dentine sampled from 32 teeth (15 from DVC and 17 from USJ), data from 477 increments were used for analysis: samples with poor collagen quality (outside the atomic C:N ratio range of 2.9–3.6) were excluded and there was some loss of samples during measurement. One sample with a C:N molar ratio of 3.2 but very high %N (30.1%) and %C (81.8%) was also rejected.

### Stable isotope measurements

For DVC, δ^15^N values range from 6.9‰ to 12.0‰ with a mean of 8.7 ± 0.9‰ and median of 8.6 ± 0.8‰, and δ^13^C values from −20.4‰ to −14.2‰ with a mean of −18.4 ± 1.0‰ and median of −18.7 ± 0.9‰. The subsampled USJ population has a range of δ^15^N values from 6.8‰ to 13.4‰ with a mean of 9.9 ± 1.1‰ and median of 9.7 ± 1.3‰, and δ^13^C values from −20.0‰ to −12.8‰ with a mean of −17.7 ± 1.5‰ and median of −18.0 ± 2.4‰.

### LEH formation age assignment

The mid-range for the LEH age assignments were used for analysis; these data can be found in the S3 Table. The results of the Wilcoxon Signed-Rank tests carried out to assess intra- and inter-observer error for the aging of LEH formation are presented in Table 5. The results of the inter-observer test suggest that there was no difference between the measured formation ages between one of the authors (ES) and the external observer. However, the intra-external observer test showed poor agreement – i.e., a difference was found in identified formation ages between the first and second round of measurements made by the external observer. The difference in intra-observer test outcomes may reflect the experience of the observers. Author ES has carried out LEH age-at-formation measurements on multiple occasions (e.g., Smith [41],), while the external observer had less familiarity with this method. The intra-observer test indicated strong agreement between the first and second rounds of measurements by ES, highlighting consistency in the identification of LEH, and replicability of LEH measurement and age assignment. The LEH formation ages assigned by the author ES were therefore used for all the following analyses.

**Table 5:** Wilcoxon signed-rank test comparing intra- and inter-observer error of estimated LEH ages.

Comparison	Wilcoxon Signed-Rank p-value
Intra-observer (ES)	0.262
Intra-observer (External)	0.007
Inter-observer	0.168

## Discussion

The co-occurrence of physiological and dietary events may confound interpretation of stable isotope data. The strong genetic control of tooth development provides some resilience against physiological stress and resistance to developmental inhibition. As a consequence, stress may result in the cessation of bone growth while tooth development continues. In such a scenario, tooth dentine δ^15^N may represent a combination of both dietary and physiological effects. During weaning, when δ^15^N values are expected to decrease, stress may result in a smaller than expected decrease, no decrease, or even an increase in δ^15^N values. Tissue recycling occurs when nutritional requirements are not being met in order to make up the difference required [62]. During these periods it is likely that the dentine values represent both current diet (regardless of sufficiency) and the value of further fractionated previous diet (from tissue recycling). Recycling of tissues seems to be a last resort, with the body preferentially using the free pool of nitrogen, therefore the fractionation is typically relative to current diet rather than that reflected in the recycled tissues [62–64]. This mix of diet and tissue recycling may lead to a variable impact on incremental dentine stable isotope values during stress.

The stress-related changes identifiable through visual-parsing of isotope scatterplots in the incremental dentine isotopic profiles of the Dugopoljie and Udbina populations had a strong and meaningful relationship with co-forming LEH (effect sizes all above 0.6). A meaningful association between LEH and conventional stress-related changes to δ^15^N was also found, further supporting a relationship between the two. Notably, the **LEH-Stress Association** of 64%/73% suggested that using stable isotope trends alone (even with the integrated context data) may leave more periods of stress unidentified (*LEH-Stress Association,* Tables 3 and 4) than when isotope trends and LEH are both considered, i.e., stress does not always manifest in a readily identifiable way in stable isotope trends. Cautiously, we suggest that this implies that LEH can be used to indicate periods of physiological stress that have likely impacted stable isotope values even where the data does not show an expected stress trend pattern, and interpretations should therefore take account of this.

The inverse correlation of δ^15^N and δ^13^C is a useful starting point for identifying stress and recovery in incremental dentine stable isotope data but, a third, independent (i.e., non-isotopic) proxy is valuable both in supporting the identification of stress in stable isotope trends and also highlighting periods of stress that may not be visible in isotope data. For example, δ^15^N values decrease during weaning, but where stress occurs concurrently the drop in δ^15^N values may not be evident or the weaning curve may look shallower than expected – the typical weaning curve being masked by stress, skewing estimations of the duration of the weaning process and potentially suggesting weaning cessation occurred later than it in reality did. DVC17 (Fig 6g), for example, had bands of LEH indicating physiological stress at 3.9 and 4.5 years and exhibited a prolonged period of higher δ^15^N values between 1.6 and 3.5 years followed by a drop in δ^15^N values, suggesting an extended period of stress but equally this could be attributed to continuation of breastfeeding. The potential pitfalls of stress obscuring dietary patterns and diachronic changes extends beyond the weaning period into childhood and adolescence when other metabolic demands during development, pregnancy and lactation can result in physiological stress [65]. Some ‘flattening’ of peaks may also be related to rapid growth (e.g., during infancy), where δ^15^N can decrease, however, the influence of growth on δ^15^N values tend to be small so the potential magnitude of the impact it would have on trends is unclear [65,66].

Published studies comparing LEH and incremental isotope trends are few in number [e.g., 43,44]. The London St Mary’s Spital medieval cemetery populations investigated by Walter *et al.* ([43]: 218–219) had an expected end-of-weaning age of between two and three years based on other studies of weaning in Medieval England. At least two individuals from the site, both with LEH (SRP98–30920 and SRP98–20682), have weaning cessation ages of around or just after three years, based on scrutiny of the published isotope data (see figs 5 and 6, Walter *et al.,* [43]). Individual SRP98–20682 has two bands of LEH, both associated with a decrease in δ^15^N values, while SRP98–30920 has one. The relatively late age at weaning of both individuals could reflect idiosyncratic variation in breastfeeding practices or health-related extension of the weaning period, e.g., failure to thrive resulting in continuation of breastfeeding. Alternatively, the appearance of late weaning may be due to a period of stress impacting δ^15^N values, which is implied by the presence of LEH. The ‘extended’ fall of δ^15^N may be due to the combined influences of weaning and tissue recycling resulting in a false impression of later weaning cessation. Crowder *et al.* [44] found similar weaning ages (c. 2.0–3.0 years) for 4^th^-7^th^ centuries CE individuals from Archiud, Romania. This included both those with and without LEH. However, the individuals without LEH had other signs of stress on the skeleton including cribra orbitalia and rickets, which may have impacted observed age at weaning estimations. While weaning age was within expected norms for the period, there are ‘interruptions’ to the decrease of the δ^15^N values that give a ‘stepped’ appearance to the weaning trend, with the ‘treads’ or flat portion of these steps correlating to LEH formation ages. The addition of LEH analysis to the evaluation of incremental dentine stable isotope data adds an extra dimension to interpretations. For example, while later weaning ages may be considered part of normal variation in practice, the identification of stress from a second proxy offers the possibility of more nuanced interpretations.

Using incremental dentine stable isotope data in isolation to assess dietary habits is of high utility where an individual has not experienced stress. During stress, however, we are ‘not what we eat’ [67]. While stable isotope values are widely acknowledged to be influenced by stress [27,29], in clinical studies of human responses to stress, the scale and direction of the changes to stable isotope ratios are not always consistent [e.g., 27,30]. Individual differences in response to stress may be due to factors such as the presence or size of fat stores or type of dietary deficiency [27,30]. The use of relief or weaning foods that have low δ^13^C values may also impact stress-related trends [29,45].

In this study, sample selection was impacted by travel restrictions during the pandemic. A key limitation acknowledged here is the small number of control samples, i.e., teeth without LEH. This was mitigated by the assessment of the correlation between stable isotopic and LEH stress indicators across divisions of the tooth crown – some of the crown portions had no LEH, thereby acting as ‘control’ areas. Moreover, the overall sample size is sufficiently large that the observed association between LEH and stress-related changes to stable isotope trends is robust.

## Conclusion

Physiological stress does not always manifest isotopically in an readily identifiable way. Variation in stable isotope trends is likely to be attributed to dietary changes, normal metabolic variation or to other physiological effects where conventional stress-related effects are not evident. Changes to stable isotopes associated with stress are anchored to diet (current diet, transitioning diet, and diet at the time of tissue formation). These factors need to be accounted for when trying to identify periods of physiological stress. Diet is also a contributor to physiological stress, particularly dietary transitions, both intentional (e.g., weaning), and not (e.g., famine). Two (or more) factors could be impacting the stable isotope ratio trends simultaneously leading to intermediate values. Therefore, stress-related changes to stable isotope trends may appear reduced if they co-occur with other processes that cause a decline in δ^15^N values (e.g., weaning or reduction in animal products in diet, both of which could also be a factor causing stress). Being able to isolate the impact of stress from diet on stable isotope values is critically important to drawing robust conclusions from incremental isotope data. This research has established a strong relationship between the presence of LEH (a physical marker, commonly associated with metabolic stress) to stress-related changes in stable isotope trends. This relationship can provide complimentary support for stress-related effects as it may not be evidenced by conventional changes in stable isotope values (increasing δ^15^N accompanied by decreasing δ^13^C). Left unidentified, important facets and interpretations of individual life histories will be lost. Evaluation, where present, of LEH formation ages, using the field method, is a straightforward process of adding resolution to the identification of stress from stable isotope ratios, and can be carried out with normal pre-processing recording of samples for destructive analysis. Recording and comparing LEH prior to incremental sampling and comparing the LEH formation ages to coeval isotopic measurement can highlight these areas of stress creating stronger/more detailed life histories and prevent misinterpretations of diet.

## Supporting information

S1 Table Observed teeth for LEH analysis. (XLSX)

S1 File Stable isotope measurement method details. (DOCX)

S1 Fig Additional incremental dentine stable isotope data overlaid with LEH formation times. Scatterplots of δ^13^C (blue circles) and δ^15^N (red squares) incremental data with bands of LEH in black. Where available adult rib stable isotope data is shown in dotted lines.
(TIF)

S2 Table Observer stress identification from stable isotope scatterplots from all tests. (XLSX)

S3 Table LEH formation ages, providing the mid-range estimated age value used for analysis. (XLSX)
