# Supplementary material for: Identification of stress from incremental dentine stable isotope trends using linear enamel hypoplasia
Source: PLoS One. 2026 Oct 9;21(10):e0359651. doi: 10.1371/journal.pone.0359651 (PMC13652263; doi:10.1371/journal.pone.0359651)
Supplement: Supplementary file 1 [file pone.0359651.s002.docx]

**S1 File: Stable Isotope Measurement**

In order to assess the employed sampling method [1] a sample-size test was designed to determine minimum sample size using homogenised dentine collagen from the remaining, post-sampling demineralised tooth. The homogenised sample was separated into several aliquots of different weight ranging from 0.1 to 1.0 mg to assess linearity effects and determine minimum sample size. Small sample weights can impact linearity and create size-dependant shifts in stable isotopes values [2]. The tests showed that samples ≥ 0.2mg were required for measurement (see S1.1 Fig). Any of the collagen samples below this weight were combined with an adjacent dentine increment. While no evidence of the linearity effect was found for the first two runs, there was a linear trend observed for the raw nitrogen isotope ratio measurements in the third run. Four samples were measured in this final run, and all were used to generate the correction equation for the linearity effect:

$$\frac{8.1734}{(\left( 0.9302*collagen weight \right)+8.1734)}*raw \delta^{15}N value$$

Only the two final batches (out of 13) of the sample run may have been impacted with the linearity issue. However, even where impacted, sample values changed only slightly and the overall trends were not impacted. See S1.1 Table and S1.2 and S1.3 Figs as an example. The lack of impact on trends was likely due to the consistent collagen weights, a larger impact may be present if there is a large range of collagen weights from the increments of one tooth.

Collagen samples were measured using a Thermo Finnigan EA IsoLink coupled to a Delta V Plus isotope ratio mass spectrometer via a Conflo IV interface. Analytical error (±1SD) calculated by repeat measurement of international standards (USGS 40 and USGS 41) was ±0.2‰ or better for carbon and ±0.3‰ or better for nitrogen isotopes. Carbon isotopes were calibrated to V-PDB with a 2 point correction using USGS 40 (-26.39‰) & USGS 41 (37.63‰) and nitrogen isotopes were calibrated to AIR with a 2 point correction using USGS 40 (-4.52‰) & USGS 41 (47.57‰) A quality check standard SADCOW a modern cow bone collagen calibrated against USGS 40 & 41 = 4.7 +/-0.04 (N), USGS 40 & 41 = -22.4 +/-0.07 (C) was used and normalisation checked using well characterised Elemental Microanalysis IRMS fish gelatin standard B2215 (δ^13^CVPDB = –22.92 ± 0.10‰, δ^15^NAIR = 4.26 ± 0.12‰).

**S1.1 Fig. Test to determine minimum sample weights (mg) required for reliable results based on the linearity effect.**

**S1.1 Table. Original and corrected (using linearity correction formula) δ^15^N values, with difference.**

| **USJ 161** | | | **DVC133B** | | |
| --- | --- | --- | --- | --- | --- |
| **Original δ^15^N** | **Linearity Corrected δ^15^N** | **Difference (Corrected-Original)** | **Original δ^15^N** | **Linearity Corrected δ^15^N** | **Difference (Corrected-Original)** |
| 12.4 | 12.2 | -0.3 | 8.8 | 9.1 | 0.3 |
| 12.5 | 12.4 | -0.2 | 8.4 | 8.6 | 0.3 |
| 12 | 11.9 | -0.2 | 8.2 | 8.5 | 0.3 |
| 12.2 | 12 | -0.2 | 8.6 | 8.8 | 0.1 |
| 12.1 | 12 | -0.1 | 7.9 | 8.1 | 0.2 |
| 11.5 | 11.5 | -0.1 | 8.1 | 8.1 | 0 |
| 11.9 | 11.8 | -0.1 | 8.3 | 8.4 | 0.1 |
| 12 | 11.9 | -0.1 | 8 | 8.2 | 0.2 |
| 12.7 | 12.7 | 0 | 9 | 9 | 0 |
| 12.4 | 12.4 | -0.1 | 9.1 | 9.1 | 0 |
| 12.7 | 12.6 | -0.1 | 9.1 | 9.3 | 0.2 |
| 12.5 | 12.4 | -0.1 | 9 | 8.9 | 0 |
| 12.8 | 12.4 | -0.4 | 9.1 | 9.1 | 0 |
| 12.8 | 12.5 | -0.2 | 9.3 | 9.4 | 0.1 |
| 12.8 | 12.7 | -0.1 | 9.3 | 9.5 | 0.2 |
| 13.1 | 12.9 | -0.2 | 9 | 9.1 | 0.1 |
| 13 | 12.7 | -0.2 | 8.7 | 8.8 | 0 |
| 13.4 | 13.4 | 0 | 8.3 | 8.3 | 0 |
|  |  |  | 8.9 | 9 | 0.1 |
|  |  |  | 9 | 9.3 | 0.3 |

**S1.2 Fig. USJ161 δ^15^N value, original (squares) vs. corrected (triangles), using small sample linearity correction.**

**S1.3 Fig. DVC133B δ^15^N value, original (squares) vs. corrected (triangles), using small sample linearity correction.**

References

1.	Czermak A, Fernández-Crespo T, Ditchfield PW, Lee-Thorp JA. A guide for an anatomically sensitive dentine microsampling and age-alignment approach for human teeth isotopic sequences. American Journal of Physical Anthropology. 2020;173: 776–783. doi:10.1002/ajpa.24126

2.	Meier-Augenstein W. Stable Isotope Forensics: Methods and Forensic Applications of Stable Isotope Analysis. 2nd ed. John Wiley & Sons, Ltd; 2018.
